# Improving Transitions of Care for Young Adults With Congenital Heart Disease: Mobile App Development Using Formative Research

**DOI:** 10.2196/formative.9963

**Published:** 2018-09-11

**Authors:** Keila N Lopez, Michael O'Connor, Jason King, James Alexander, Melissa Challman, Donna K Lovick, Nicole Goodly, Amelia Smith, Elliott Fawcett, Courtney Mulligan, Debbe Thompson, Michael Fordis

**Affiliations:** 1 Division of Pediatric Cardiology Department of Pediatrics Baylor College of Medicine/Texas Children's Hospital Houston, TX United States; 2 Center for Collaborative and Interactive Technologies Baylor College of Medicine Houston, TX United States; 3 Children's Nutrition Research Center US Department of Agriculture, Agricultural Research Service Baylor College of Medicine Houston, TX United States

**Keywords:** adolescent health, chronic disease, transitions of care, health disparities, mobile health, mHealth, patient empowerment, patient involvement, self-efficacy, user-centered design

## Abstract

**Background:**

Congenital heart diseases (CHDs) are the most common type of birth defects. Improvements in CHD care have led to approximately 1.4 million survivors reaching adulthood. Successful transition and transfer from pediatric to adult care is crucial. Unfortunately, less than 30% of adolescents with CHD successfully transition to adult care; this number is lower for minority and lower socioeconomic status populations. Few CHD programs exist to facilitate successful transition.

**Objective:**

The goal of our study was to describe the formative research used to develop a prototype mobile app to facilitate transition to adult care for adolescents with CHD.

**Methods:**

A literature search about best practices in transition medicine for CHD was conducted to inform app development. Formative research with a diverse group of CHD adolescents and their parents was conducted to determine gaps and needs for CHD transition to adult care. As part of the interview, surveys assessing transition readiness and CHD knowledge were completed. Two adolescent CHD expert panels were convened to inform educational content and app design.

**Results:**

The literature review revealed 113 articles, of which 38 were studies on transition programs and attitudes and 3 identified best practices in transition specific to CHD. A total of 402 adolescents aged 15 to 22 years (median 16 years) participated in semistructured interviews. The group was racially and ethnically diverse (12.6% [51/402] African American and 37.8% [152/402] Latino) and 42.0% (169/402) female; 36.3% (146/402) received public insurance. Most adolescents (313/402, 76.7%) had moderate or severe CHD complexity and reported minimal CHD understanding (79.0% [275/348] of those aged 15 to 17 years and 61.1% [33/54] of those aged 18 to 22 years). Average initial transition readiness score was 50.9/100, meaning that transition readiness training was recommended. When participants with moderate to severe CHD (313/402, 77.9%) were asked about technology use, 94.2% (295/313) reported having access to a mobile phone. Interviews with parents revealed limited interactions with the pediatric cardiologist about transition-related topics: 79.4% (331/417) reported no discussions regarding future family planning, and 55.2% (230/417) reported the adolescent had not been screened for mental health concerns (depression, anxiety). Further, 66.4% (277/417) reported not understanding how health care changes as adolescents become adults. Adolescents in the expert panels (2 groups of 3 adolescents each) expressed interest in a CHD-specific tailored app consisting of quick access to specific educational questions (eg, “Can I exercise?”), a CHD story-blog forum, a mentorship platform, a question and answer space, and a checklist to facilitate transition. They expressed interest in using the app to schedule CHD clinic appointments and receive medication reminders. Based on this data, a prototype mobile app was created to assist in adolescent CHD transition.

**Conclusions:**

Formative research revealed that most adolescents with CHD had access to mobile phones, were not prepared for transition to adult care, and were interested in an app to facilitate transition to adult CHD care. Understanding adolescent and parent needs, interests, and concerns helped in the development of a mobile app with a broader, tailored approach for adolescents with CHD.

## Introduction

Congenital heart diseases (CHDs) are the most common type of birth defects, observed in 40,000 babies born in the United States each year [1,2]. Improvements in the care of those with severe CHD have lead to a decline in childhood CHD mortality over the last 20 years [3], with roughly 1 million survivors now reaching adulthood [4-8]. This emerging survivor population requires lifelong surveillance and disease management, as patients are often palliated but not cured [9], putting them at risk for substantial morbidity and mortality and placing a large burden on health care resources [10].

While it is critical that CHD patients suffer no lapses in cardiac care, this is often not the case. A further concern is that lapses in CHD care appear to be a predictor for morbidity [11]. Disparities in the medical care provided to the growing CHD adolescent survivor population involve poor care transition (an age and developmentally appropriate process addressing the medical, psychosocial, and educational and vocational aspects of care) from child-centered to adult-centered health care [8,12] and lack of appropriate transfer of care (the point at which an adult cardiac provider assumes the medical care of a CHD patient) [1,2,12-14]. Lack of assessment of transition readiness (capacity of the adolescent and medical team to initiate and successfully complete the transition process) [15,16] compounds disparities in quality care [17]. Disparities become further magnified in ethnic minorities [4-8,13,18]. Beyond health care access, disease knowledge, and transition readiness, studies show a mentoring relationship [19-21] for adolescents with chronic disease is crucial for successful transition to adult care [22].

The transition period is a vulnerable time for adolescents with CHD, and many drop out of active health care at this time. This leads to poor health outcomes and impedes transfer to adult care [23,24]. Improving transition and transfer are critical to successful long-term disease management and survival. In the clinical setting, providers often do not have the time or resources to address the educational and preparatory needs of transitioning adolescents. One study surveying CHD providers reported that 31% of them feel that adolescents are adequately prepared for transition. When asked about barriers to their involvement in the transition process, 69% of providers stated lack of a structured transition program and 56% stated lack of time in clinic [25]. In the clinical setting, if a general resource for transition is provided, it is often a nontailored educational handout about CHD and does not address transition readiness or social support needs during this high-risk period. To address these important gaps in care, the needs and concerns of all transitioning adolescents with CHDs should be identified and addressed beyond what is available in clinic.

Patient-centered self-management programs (clinical and online) have shown improvements in adults with chronic disease across health status measures, healthy behaviors, and self-efficacy as compared to usual care [26,27]. Of the chronic disease patient-centered mobile apps that currently exist, most target daily management of medications and symptoms for conditions like diabetes and asthma [28]. While there is little evidence regarding patient self-management e-based programs for care transition in adolescents, there is evidence that young adults have an interest in internet- and mobile-based programs for chronic disease management [29-31]. Many adolescents have ready access to mobile technology; 1 in 4 adolescents are cell-mostly internet users, 78% of adolescents have a mobile phone, and nearly half (47%) of those own smartphones [32]. Mobile phone access is particularly high for minorities. In Latinos aged 18 to 29 years, more (66%) own a smartphone than a computer (34%) [33]. Additionally, 72% of all African Americans and 98% of 18- to 29-year-olds in the group have either a broadband connection or a mobile phone [34]. A 2017 study found that African American adolescents are more active on social media and messaging apps than their white peers [35]. Historically, black adolescents report greater mobile phone use than white adolescents and are now more likely to use social media platforms optimized for mobile phones.

Prior research was conducted with adolescents and adults with CHD before and after their transition to identify their preferred methods for learning about CHD care transition. Findings revealed that while patients preferred to learn about their condition and long-term implications of their CHD directly from their physicians, they were also receptive to Web-based modalities to obtain similar information important to the success of adolescent transition [36]. Nine out of 10 adolescents used their mobile phone to access the internet, and 100% of those sent text messages daily. They were uniformly interested in a mentorship program with adults who have CHD and were interested in interacting with other CHD adolescents [36]. This research proposed that there were other ways, beyond face-to-face with a physician, that adolescents were willing to learn about their CHD and gain transition skills. It also demonstrated that the majority of CHD adolescents had ready access to mobile phones regardless of socioeconomic status. This suggests that adolescents with CHD would be receptive to a mobile app for the purpose of CHD knowledge and transition.

## Methods

### Project Overview

Our research was conducted to collect formative data to inform the content and structure of a mobile app that facilitates transition and transfer of adolescents with CHD to adult care. It was conducted as part of (1) a quality improvement project for adolescents with mild-to-severe CHD to improve their experience when transitioning from a pediatric cardiologist to an adult cardiologist and (2) a research study to inform the creation of a mobile app (principal investigator Julie Miller, U24HL13569 and principal investigator KNL, K23HL127164). The project was based on the Got Transition website [37], the American Heart Association’s best practices for transitioning adolescents with CHD [13], and an ongoing randomized controlled trial for transitioning adolescents with CHD [10]. The formative research included a literature review, parent and adolescent interviews, and discussions with 2 expert panels.

### Setting

The formative research was conducted in a hospital-based pediatric cardiology clinic serving patients with CHD in the southwestern United States. The clinic services over 2000 adolescents with CHD per year. The clinic also has an adult congenital component that services young adults with congenital heart disease. The quality improvement project was initiated to improve transition readiness and transfer of care from the pediatric to adult congenital cardiologists. Prior to the project, there was no formal transition process, which put patients at risk for being lost to follow-up and having gaps in care upon being discharged from the pediatric cardiology clinic.

This quality improvement project began in April 2016 and had several iterative cycles to determine clinic flow, create a standardized transition-based CHD educational curriculum, and determine adolescent CHD patient needs and preferences and identify gaps regarding transition-related education. The research project began to build the mobile app in September 2015, and it was revised based on patient data surrounding knowledge and transition readiness deficits. This research was approved by the Human Subjects Review Committee at Baylor College of Medicine (H-39154).

### Literature Review

A literature review was conducted to determine best practices and guidelines in transition medicine for CHD adolescents. For our search methods, we searched PubMed for papers published from January 2001 through November 2017. Our search terms included transition, congenital heart disease, and adolescents. We considered articles that described best practices, guidelines, expert opinion, literature reviews, transition programs, and surveys exploring attitudes surrounding transition for adolescents with CHD.

### Interviews

#### Selection Criteria

Inclusion criteria were adolescent patients aged 15 to 21 years with congenital heart disease or electrophysiologic abnormalities who were being seen for a scheduled appointment in our pediatric cardiology clinic. Exclusion criteria included adolescent patients who had a known genetic or chromosomal disorder or a significant developmental delay.

#### Recruitment

Based on a quality improvement initiative conducted at our institution to improve transition in the pediatric cardiology clinic, we approached parents and patients prior to their cardiology clinic visit to determine if they wanted to participate in our study. Beginning April 2016, patients were identified by searching weekly clinic schedules for patients meeting the inclusion criteria. Prior to the clinic visit, pediatric cardiology providers were contacted by a transition nurse and social worker to determine if there was any hesitation to initiating transition in patients meeting inclusion criteria. Parents and adolescents were then approached in the waiting room by a transition nurse or social worker to determine interest in participating in the quality improvement project. Parents and adolescents were then given an introduction and explanation regarding the program; if they were interested in participating, they were offered an information packet about our transition program and scheduled to formally see the transition team on their the next clinic visit with the pediatric cardiologist. If they were not interested in the program, they could opt out of the program at the time of introduction.

#### Procedure

A standardized script guided discussions with both adolescents and their parents. Sample questions included “Can you name your congenital heart disease?” and “Do you know your medications and why you are on them?” Semistructured private interviews were conducted by members of the transition team (transition nurse, social worker) trained in qualitative methods. Adolescents and parents were interviewed separately during the clinic visit between medical procedures (echocardiogram, electrocardiogram, cardiologist visit).

#### Adolescents

Parents of adolescents with CHD were asked to leave the clinic room while adolescent interviews took place. Adolescent demographics, CHD type, access to mobile technology, and transition-specific topics were asked by the interviewer using a standardized script developed during the quality improvement process. Responses were manually recorded by the interviewer. CHD knowledge and motivation were also assessed by asking the adolescent to complete a paper version of a draft knowledge questionnaire that we developed as a part of the quality improvement initiative. Adolescents completed a paper version of the Transition Q survey [38] to assess transition readiness.

#### Parents

Parents left the clinic room where adolescents were being interviewed and were taken to a separate room in the clinic where they were interviewed. The interviewer asked parents about their interest in having their child begin the transition process, communication with pediatric cardiologists regarding transition topics, familiarity with the transition process, and systemic changes that occur as their children become adults (insurance, advanced directives, etc). Sample questions included “Does your child have a plan to keep their health insurance after age 18 years?” and “Has your pediatric cardiologist ever discussed the topic of advanced directives with you?”

Data from all interviews and self-report questionnaires were manually entered into a Research Electronic Data Capture (RedCAP) database by the transition team for the iterative process.

#### Expert Panel

Adolescents with CHD who expressed an interest in mobile app development during their interview were contacted by phone to determine interest in being a part of an expert panel. Adolescents agreeing to participate in the expert panel were asked to assist more specifically in the development of the mobile app. Qualitative data were obtained in an unstructured group interview conducted by the transition team director and nurse. Panel members were asked their feelings and thoughts about what should be included on a CHD mobile app about transition. Manual notes were taken by the transition social worker who was not participating in the interview, and these were subsequently coded into themes. Adolescents were shown several images of the prototype mobile app on a tablet device to determine their general impression of the design and appearance of the app. Data from these interviews were subsequently manually entered into RedCAP.

#### Prototype

The mobile app structure was created based on best practice guidelines, our formative research, and CHD expert panel suggestions. An iterative, user-centered design process was used to ensure that the design and functionality of the app and content were compatible with the target population’s needs and preferences. The app was structured with a client-server architecture, with all user data retained on the server to facilitate secure data storage and transfer.

## Results

### Literature Review

A total of 113 journal articles from the United States and Europe addressing transition were identified. Of these, 38 were studies on transition programs and attitudes surrounding transition. Three articles gave best practices and guidelines in transition specific to CHD [13,38,39]. Transition best practices included timing of transition, establishing a medical home, long-term medical and surgical follow-up, anticipatory guidance, exercise restrictions, family planning, and insurance preparation. Information on transition-related topics, best practices in transition goals, and attitudes about existing transition programs were then considered for incorporation into our mobile app.

### Interviews

#### Adolescents

##### Demographics

We completed 402 individual interviews with adolescents who had CHD (Table 1). Adolescents were aged 15 to 22 years (median age 16 years; interquartile range [25% to 75%) 14 to 17 years). Interviews lasted an average of 18 to 22 minutes.

##### Mobile Phones

Of the patients asked, 94.2% (295/313) of the adolescents had their own mobile phone, with 67.8% (200/295) of those being iPhones and 32.2% (95/295) having Android operating systems. A total of 23 adolescents were interested in mentoring other adolescents with CHD.

##### Congenital Heart Disease Knowledge

Most adolescents with all levels of CHD complexity reported minimal to no understanding of their CHD (79.0% [275/348] of those aged 15 to 17 years and 61.1% [33/54] of those aged 18 to 22 years).

##### Transition Readiness

Average initial transition readiness for all adolescents was 50.9 out of 100. Thus, roughly half of the transition readiness metrics were being met by adolescents during the transition period.

#### Parents

We conducted independent interviews with parents of adolescents with CHD; 89.7% (374/417) of parents felt that their child was ready to start learning about and participating in the transition process, and 89.0% (371/417) reported understanding the need for eventual transition and transfer to an adult CHD provider. One in 5 (86/417, 20.6%) parents required an interpreter. Only 5.0% (21/417) of parents reported that the pediatric cardiologist saw their child independent of them. More than three-quarters (331/417, 79.4%) of parents reported that the pediatric cardiologist had not addressed family planning or birth control, 55.2% (230/417) reported never having received a question about the mental health of their adolescent (ie, no questions about depression or anxiety), and 81.5% (340/417) of parents reporting that the pediatric cardiologist had not addressed the topic of advanced directives.

Finally, 63.8% (266/417) of parents had not discussed anticipating independent decision making by their child after age 18 years, 66.4% (277/417) of parents reported not understanding how health care may change after age 18 years, and 40.8% (170/417) reported not understanding what happens to their child’s insurance after age 18 years.

#### Expert Panel

On initial interview, 10 adolescents expressed an interest in mobile app development. These adolescents were contacted, and 6 expressed an interest in being a part of an advisory council to assist in the development of a CHD mobile app. In 2 expert panels of 3 adolescents each, 4 themes arose around main areas desired in the mobile app, along with specific recommendations for topics to include (Textbox 1). Quotations addressing distinct design components of the mobile app for each theme can be seen in Textbox 2.

### Mobile Prototype

**Figure 1:**
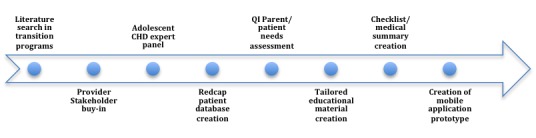
Flow diagram from concept to mobile app creation. CHD: congenital heart disease; QI: quality improvement.

A summary from concept to creation of mobile app is shown in Figure 1. The server component was built in an environment compliant with the Health Insurance Portability and Accountability Act and the Federal Information Security Management Act within Amazon Web Services that included administrative and reporting features in addition to a JavaScript Object Notation–based Representational State Transfer application programming interface used by the client application. Patient data (including portable health summary documents and other structured protected health information) was stored in encrypted storage and decrypted only when required for delivery to an authenticated and authorized client, at which time it was delivered via a secure, encrypted channel.

**Figure 2:**
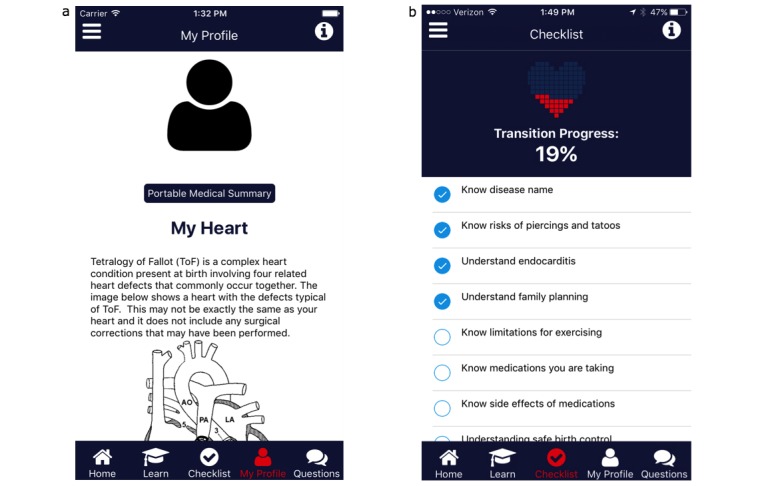
Screenshots of the mobile app: (a) profile page with congenital heart disease diagram and medical summary and (b) congenital heart disease transition checklist.

**Figure 3:**
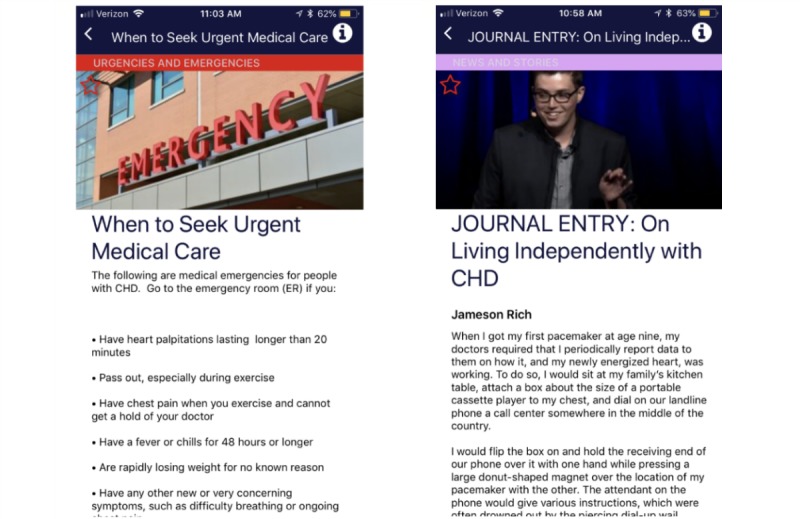
Transition education on congenital heart disease medical emergencies and blog entry on life with congenital heart disease for mobile app. CHD: congenital heart disease; ToF: Tetralogy of Fallot.

**Figure 4:**
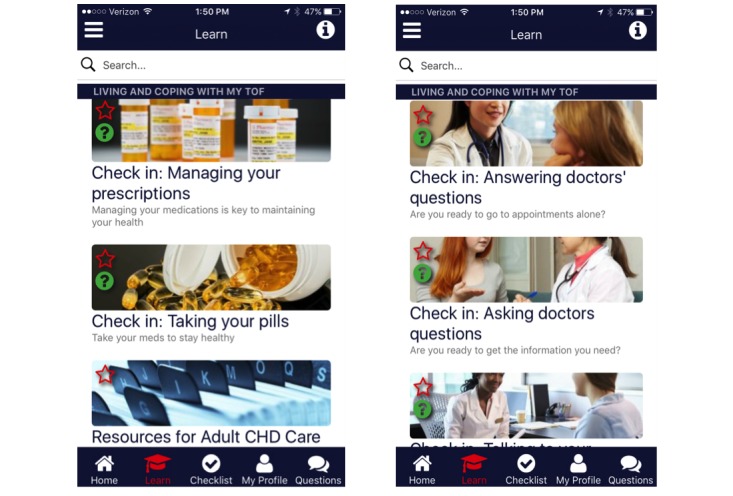
Congenital heart disease (CHD) in-clinic transition skills, out-of-clinic transition skills, and adult resources for mobile app. ToF: Tetralogy of Fallot.

**Figure 5:**
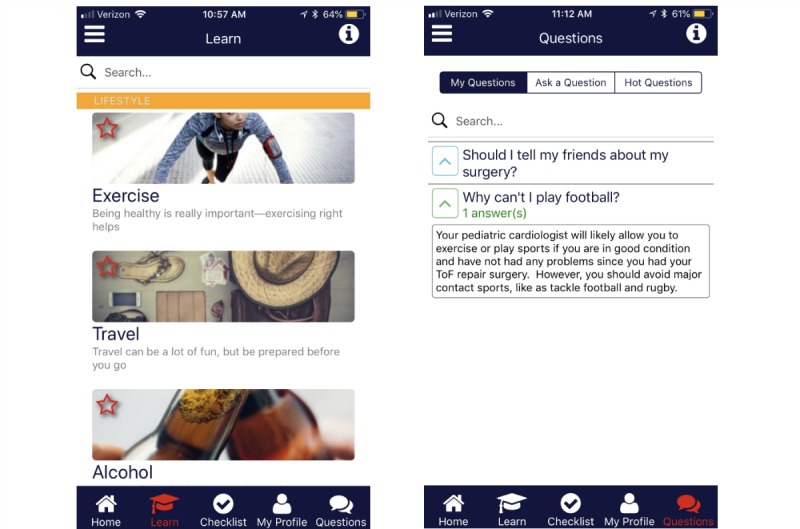
Transition education on congenital heart disease (CHD) aspects of lifestyle and message board for CHD related questions.

The client application was a native iOS application built using the React Native framework. This technology was chosen for its efficient development cycle and support for cross-platform deployment capability (the potential for deployment to both Android and iOS devices with minimal platform-specific development). Users could authenticate to the server by providing a username and password in the app. Future new users of the app could use a 1-time activation code to establish authentication credentials during their first time using the app. Mobile app prototype included user profile, transition checklist component, CHD-specific educational modules, portable medical summary and CHD diagram, a space for medical questions, and a blog space (Figures 2-5).

**Table 1:** Characteristics of patients with congenital heart disease on the expert panel making recommendations for mobile app.

Characteristics	Total, n (%)
Sex (female)	169 (42.0)
Age (15-17 years)	348 (86.5)
Race (African American)	51 (12.6)
Ethnicity (Latino)	152 (37.8)
Public insurance	146 (36.3)
Moderate/severe complexity congenital heart diseases	313 (76.7)

**Themes and recommendations for the mobile app.**

Tailored application:

- Create a congenital heart disease (CHD) diagram specific to CHD patient
- Enable a transition checklist
- Create way to monitor transition progress
- Create portable medical summary
- Create medication and appointment notifications

CHD-specific education:

- Focus on CHD education that is disease specific
- Make learning topics searchable
- Create short CHD videos
- Focus on how to recognize an emergency
- Do not make people take a full quiz

Mentorship:

- Design a way to connect with others who have CHD
- Connect with peer mentors
- Adult CHD mentor access
- Enable chat features with mentors

Social media:

- Create a CHD story blog for adolescents to share their experiences
- Create forum to ask medical questions
- Create way to comment on other CHD posts

**Themes and quotations addressing distinct design components for the mobile app. CHD: congenital heart disease.**

Tailored application:

- “I like assessment tailored to me...it shouldn’t be too long or I’ll just click anything.” [female]
- “I would like a personalized medical summary.” [male]
- “I prefer one-on-one education versus learning in a group.” [multiple similar quotes]
- “I would like an app aspect of being able to ask a question anonymously.” [anonymous]

CHD-specific education:

- “I knew I had surgery but did not know specifics of my CHD, and life has been worrisome.” [female]
- “I don’t want a lecture, an hour is too long. Fifteen to thirty minutes max.” [male]
- “A checklist would be helpful in knowing where I am as far as my knowledge about my CHD; seeing it helps me remember.” [multiple similar quotes]
- “Between age 13 or 14 is when I really became curious about what I had.” [anonymous]

Mentorship:

- “Pairing someone who is comfortable with their CHD to someone who isn’t comfortable with their CHD is important.” [female]
- “I would like a learning night; a good way to be social with others and learn.” [male]
- “I don’t know what questions to ask my doctor about my future, but I want to learn.” [multiple similar quotes]
- “I feel like I need knowledge and a CHD community.” [anonymous]

Social media:

- “I think it’s important to connect with others with CHD and without CHD.” [female]
- “I’d like to connect with people with similar CHD stories.” [multiple similar quotes]

## Discussion

### Principal Findings

There are increasing numbers of adolescents with chronic conditions surviving to adulthood and needing adult-centered care, and significant declines in treatment adherence are observed during the transition period [40]. Given that CHD is the most common birth defect and there are now more adults surviving than children, empowering adolescents to appropriately transition care is of utmost importance. Personal management of chronic physical conditions requires 5 main skills: problem solving, decision making, resource utilization, patient-doctor relationships, and taking action [41]. This prototype mobile app can assist in aiding problem solving and decision making by providing disease-specific and lifestyle information and creating portable tools, including a portable medical summary and a CHD diagram of their disease, that adolescents can use to convey important disease information.

The formative research with adolescents and their parents provided insight into the transition domains that adolescents with CHD seem to be lacking in terms of education during routine clinic visits with their pediatric cardiologist. These domains include more precise CHD knowledge, medication knowledge, lifestyle choices, long-term care, and family planning, and we have directly integrated these into a mobile app created to assist in the transition of care for adolescents with CHD.

Several health-related mobile apps exist for adults with a variety of chronic diseases. A 2015 systematic review of studies of adolescent use of mobile apps supporting management of chronic disease revealed only 4 studies that contained pre-post or randomized controlled data [42]. Of the 4 apps studied, 2 were focused on type 1 diabetes and the other 2 focused on asthma and cancer. Three out of the 4 studies reported some level of patient involvement in the design, development, or evaluation of the app. This review noted that the dearth of studies and overall small sample size emphasized the need for future studies on the development, use, and effectiveness of mobile apps to support adolescent personal management of chronic disease [42]. There is no mobile app currently available, to our knowledge, for assisting patients with CHD in the transition process or in managing their disease. Thus, our mobile app fills a gap for a high-risk, heterogeneous population that requires lifelong management and care of their disease.

Two other systematic reviews were conducted in 2017 looking at using mobile phone apps and text messaging to improve medication adherence in adolescents with chronic diseases [43] and improve preventative behavior in adolescents [44]. The first review found that 7 out of 15 studies meeting inclusion criteria demonstrated significant improvement in adherence with moderate to large standardized mean differences. The second review found 19 studies meeting the inclusion criteria for promoting preventative behaviors, with 4 of those studies involving mobile apps and 15 involving only text messages. About half of the included studies (8/19) demonstrated significant improvement in preventative behaviors with moderate standardized mean differences.

Given that some of our domains are adherence-related and others are preventative, this is encouraging news for the potential impact our mobile app can make. That being said, the evidence to support the cost effectiveness of text messaging and mobile phone–based interventions in improving medication adherence in adolescents with chronic health conditions is insufficient. More research needs to be done in this area to better understand their role in cost savings while improving medication adherence and health outcomes [45].

Next steps include surveying pediatric and adult cardiologists on the content in the mobile app and conducting focus groups on the existing prototype of the app to obtain feedback on the design, interface, and information included in the mobile app.

### Limitations and Strengths

Our formative research was performed in the context of a quality improvement project, and as the project changed our practice to address clinical needs, our parent and patient intake changed (so not all questions were asked to all parents and patients). The evolution of the quality improvement project along with the expert panel then helped inform our mobile app.

For our quality improvement project, we excluded patients with chromosomal abnormalities, genetic syndromes, or developmental delays. Therefore, parental attitudes and concerns for those populations were not reflected in parental interviews. This may limit the generalizability of a CHD transition mobile app for these populations.

One strength of our formative research reported here is that it was conducted with adolescents from different socioeconomic levels and races/ethnicities. These adolescents also had differing levels of disease severity, making our formative research informative for all patients with CHD. We are hoping this assists in the acceptance, usability, and adoption of the mobile app.

### Conclusions

Given the limitations most providers face in terms of resources and time to address and teach about transition topics, the purpose of this paper was to report formative research conducted to inform the development of a mobile app to facilitate the transition and transfer care of adolescents with CHD to adult care.

This research revealed knowledge gaps and lack of transition readiness for CHD adolescents and their parents surrounding the transition process. Parents were largely interested in initiating the transition process with their adolescent and noted several areas specific to transition that were not addressed during pediatric cardiology visits. This research also revealed that in a racially, ethnically, and socioeconomically diverse population of CHD patients, the majority had access to mobile phones. Adolescents expressed interest in a mobile app to facilitate transition to adult care and had specific content areas that they wanted included in the mobile app. Future directions include a further query of pediatric cardiology providers and usability and feasibility testing with the prototype CHD mobile app.

## Abbreviations

CHD: congenital heart disease
RedCAP: Research Electronic Data Capture
